# Mechanisms Responsible for Larval Diapause in *Anastatus japonicus* Ashmead, Shown by Integrated Transcriptomic and Proteomic Analyses

**DOI:** 10.3390/insects17030306

**Published:** 2026-03-11

**Authors:** Junjian Xiao, Yi Guo, Zixin Liu, Xiaoxia Xu, Baoxin Zhang, Dunsong Li, Can Zhao

**Affiliations:** 1 Plant Protection Research Institute, Guangdong Academy of Agricultural Sciences, Key Laboratory of Green Prevention and Control on Fruits and Vegetables in South China Ministry of Agriculture and Rural Affairs, Guangdong Provincial Key Laboratory of High Technology for Plant Protection, Guangzhou 510640, China; 18219433054@163.com (J.X.); guoyi20081120@163.com (Y.G.); 15581000722@163.com (Z.L.); zhbx325@139.com (B.Z.); lidunsong@gdaas.cn (D.L.); 2 National Key Laboratory of Green Pesticide, College of Plant Protection, South China Agricultural University, Guangzhou 510642, China; xuxiaoxia111@scau.edu.cn

**Keywords:** *Anastatus japonicus* Ashmead, larval diapause, RNA-seq, proteome analysis

## Abstract

Diapause enables insect survival in unfavorable environments. The parasitic wasp *A. japonicus*, a natural enemy of several fruit-tree pests, undergoes larval diapause. Previous work has shown that larval diapause in *A. japonicus* can be induced by medium temperatures in combination with short-day photoperiods; however, the molecular functions associated with this response remain poorly understood. Here, integrated transcriptomic and proteomic approaches were employed to investigate the molecular signatures associated with larval diapause in *A. japonicus*. The identification of 3399 differentially expressed genes and 3112 differentially expressed proteins was carried out between diapause and non-diapause larvae. Among these, five gene–protein pairs showed consistent differential expression, including farnesol dehydrogenase, crystallin (associated with longevity-related pathways), forkhead-associated (FHA) domain-containing proteins, and the detoxification enzyme cytochrome P450. These findings show that larval diapause in *A. japonicus* is accompanied by extensive physiological and biochemical remodeling, and juvenile hormone-related signaling is likely involved in this process. In summary, these results provide insights into future gene function research, especially with regard to the mechanism of larval diapause in *A. japonicus*.

## Simple Summary

Diapause is a developmental arrest induced by unfavorable environmental conditions and involves coordinated physiological and biochemical adjustments, governed by multiple interacting regulatory pathways. In parasitoid wasps, which serve as important natural enemies of agricultural pests, diapause functions as an adaptive strategy to extend the storage period of biological control products. However, despite its applied relevance, the molecular mechanisms controlling larval diapause in *Anastatus japonicus* remain poorly understood. In the present study, integrated transcriptomic and proteomic analyses were performed to compare diapausal and non-diapausal mature larvae. Five candidate genes were identified as potential regulators of the diapause process. The results offer insight into the molecular foundation of diapause in *A. japonicus*.

## 1. Introduction

Diapause is a developmentally arrested state triggered by adverse environmental conditions and characterized by complex physiological and biochemical changes regulated by multiple interacting mechanisms [1,2,3]. Moreover, diapause may be divided into two categories, namely, facultative, which is triggered by environmental factors, particularly temperature and photoperiod, and is frequently seen in multivoltine species where both diapausing and non-diapausing individuals coexist, and obligatory, which happens consistently in every generation as a fixed overwintering strategy independent of environmental cues [4,5,6]. In nature, species such as *Lymantria dispar* and *Leguminivora glycinivorella* undergo obligatory diapause at the egg and larval stages to survive winter conditions. Conversely, *Helicoverpa armigera* shows facultative diapause, with short photoperiods significantly enhancing diapause intensity [7,8,9]. Similarly, in the parasitoid wasp *Anastatus japonicus*, a photoperiod of 10L:14D at 17 °C has been shown to induce the highest diapause incidence at the larval stage [10].

As important natural enemies, parasitoid wasps can utilize diapause as a special strategy to prolong the shelf life of biocontrol products [11]. *Tessaratoma papillosa*, *Halyomorpha halys*, and *Riptortus pedestris* are just a few of the agricultural pests that *A. japonicus* parasitizes as an essential egg parasitoid [12,13,14,15]. Currently, diapause induction is successfully used for the long-term storage and mass rearing of *A. japonicus* using eggs of the factitious host *Antheraea pernyi* [10,16].

The molecular regulatory mechanisms underlying larval diapause in *A. japonicus* remain largely unknown, despite its practical significance. Understanding these mechanisms is essential for optimizing mass production and improving the efficacy of biocontrol applications. High-throughput transcriptomic and proteomic technologies have proven effective in revealing the molecular basis of diapause across various insect species [17,18,19]. In *Laodelphax striatellus*, for example, RNA-seq (Ribonucleic acid sequencing) and proteomic analyses identified key genes and proteins involved in nymphal diapause, including the regulatory transcription factor *LsFoxO* [20,21]. Similar analyses in *Chrysoperla nipponensis* uncovered candidate genes and ten proteins potentially regulating adult reproductive diapause [4,22].

Here, mature diapausing and non-diapausing *A. japonicus* larvae were subjected to integrated transcriptome and proteomic analysis. Five important genes were identified as possibly involved in diapause control. The results obtained provide important new insights into the molecular mechanisms underlying *A. japonicus* diapause, setting the stage for further gene function research and the development of long-term biocontrol methods.

## 2. Materials and Methods

### 2.1. A. japonicus Rearing and Sample Preparation

*A. japonicus* was supplied by the Institute of Plant Protection of the Chinese Academy of Agricultural Sciences, Ministry of Agriculture and Rural Affairs, CABI Biosafety Joint Laboratory (CABI, Beijing, China). Eggs from the brown marmorated stink bug (*Halyomorpha halys*) with parasitic *A. japonicus* were acquired from a field setting in the suburbs of Beijing, China, and were used to create the first *A. japonicus* colony. Using unfertilized *Antheraea pernyi* eggs as a substitute host, *A. japonicus* colonies were kept in 32 × 25 × 9 cm plastic boxes under controlled conditions (24 °C, 70% relative humidity, 16 L: 8D cycle). Adult *A. japonicus* were fed honey.

After parasitic invasion for two days in a laboratory setting, *Antheraea pernyi* eggs were either placed in an environment conducive to normal development (24 °C, 16L:8D) for twelve days (non-diapause mature larvae) or in an environment favoring diapause (17 °C, 10L:14D) for forty-five days (diapause mature larvae).

### 2.2. Protein Extraction and Quantification

After grinding in liquid nitrogen, samples were lysed in 100 mM NH_4_HCO_3_ (pH 8) with 8 M urea, and 0.2% SDS, ultrasonicated (5 min, on ice), and centrifuged (12,000× *g*, 15 min, 4 °C). Proteins in the supernatant underwent reduction with 10 mM dithiothreitol (1 h, 56 °C) and subsequently alkylation with iodoacetamide (1 h, ambient temperature, away from light). Proteins were precipitated by vortexing in 4 times the volume of the pre-cooled acetone, followed by incubation at −20 °C for a minimum of 2 h and centrifugation. The pelleted proteins were rinsed twice with chilled acetone and then redissolved in a buffer with 6 M urea and 0.1 M triethylammonium bicarbonate (TEAB, pH 8.5).

### 2.3. TMT Labeling and LC-MS/MS

For each sample, 120 μg of protein was made up to 100 μL with dissolution buffer. Following the inclusion of trypsin (1.5 μg) and 100 mM TEAB buffer (500 µL), the mixture was maintained at 37 °C for 4 h for initial digestion. After adding an extra 1.5 μg of trypsin and CaCl_2_, the samples were digested overnight. After digestion, the mixture was subjected to centrifugation (12,000× *g*, 5 min, ambient temperature) after formic acid was included to acidify the solution to pH < 3. After loading the resultant supernatant onto a C18 column for desalting, three rinses with washing buffer including 0.1% formic acid and 3% acetonitrile, the material was eluted using 70% acetonitrile and 0.1% formic acid, followed by lyophilization of the eluates. After reconstituting the dried peptides in 100 μL of 0.1 M TEAB buffer, 41 μL of TMT in acetonitrile was introduced with gentle shaking at ambient temperature for 2 h and the addition of 8% ammonia for quenching. Equal amounts of samples were then mixed, desalted, and lyophilized.

An EASY-nLC^TM^ 1200 UHPLC system (Thermo Fisher Scientific, Waltham, MA, USA) connected to a Q Exactive HF-X mass spectrometer (Thermo Fisher Scientific) running in data-dependent acquisition (DDA) mode was used to build the transition library for shotgun proteomics investigations. A self-packed C18 nano-trap column (2 cm × 75 μm, 3 μm particle size) was filled with around 1 μg of each sample. As shown in Table 1, peptide separation was accomplished using a linear gradient on a self-packed analytical C18 column (15 cm × 150 μm, 1.9 μm particle size). The Q Exactive HF-X mass spectrometer (Thermo Fisher Scientific, Waltham, MA, USA), equipped with a Nanospray FlexTM electrospray ionization source and operating at a spray voltage of 2.3 kV and a capillary temperature of 320 °C, was used to analyze the eluted peptides. Complete MS scans were obtained at a 60,000 (at *m*/*z* 200) resolution over a range of 350–1500 *m*/*z*, with a maximum injection duration of 20 ms and an automated gain control (AGC) target of 3 × 10^2^. Higher-energy collisional dissociation (HCD) and MS/MS analysis were conducted on the 40 most energetic precursor ions from each complete scan. For 6-plex TMT studies, fragment ion spectra were captured at 30,000 resolution (at *m*/*z* 200), with a 5 × 10^4^ AGC target, 54 ms maximum injection duration, 32% normalized collision energy,1.2 × 10^5^ intensity threshold, and 20 s dynamic exclusion duration.

### 2.4. Data Analysis

Proteome Discoverer version 2.2 (PD 2.2; Thermo Fisher Scientific) was utilized to independently search the LC-MS/MS (Liquid Chromatography-Tandem Mass Spectrometry, Thermo Fisher Scientific, Waltham, MA, USA) spectra generated by individual runs against the *A. japonicus* protein database using a 10 ppm mass tolerance of precursor ions and a fragment ion mass tolerance of 0.02 Da. Methionine oxidation and TMT labeling were designated as variable modifications, while carbamidomethylation was designated as a permanent modification. Furthermore, in PD 2.2, acetylation and TMT labeling were considered N-terminal changes. Tryptic cleavage sites could be missed up to twice.

To ensure high-confidence identification, the data were also filtered within PD 2.2. Only peptide spectrum matches (PSMs) with confidence levels > 99% were accepted, with at least one unique peptide required per protein. Both PSMs and proteins were retained only at false discovery rates (FDRs) ≤ 1.0%. Protein data were analyzed using *t*-tests. Differentially expressed proteins (DEPs) between the groups were determined using the criteria of fold change (FC) ≥ 2.0 and *p* ≤ 0.05 for upregulation and FC ≤ 0.50 and *p* ≤ 0.05 for downregulation.

### 2.5. RNA Isolation, Quality Qualification, and RNA Sequencing

Total RNA was isolated using TRIzol (TIANGEN, Beijing, China) as directed, and was assessed using a 2100 Bioanalyzer (Agilent Technologies, Santa Clara, CA, USA) and an RNA Nano 6000 Assay Kit, before use for library construction.

Poly (T) oligo-conjugated magnetic beads were utilized to separate mRNA from total RNA to prepare an RNA-seq library. The purified mRNA was fragmented in First Strand Synthesis Reaction Buffer (5×) under elevated temperature in the presence of divalent cations. Random hexamer primers and M-MuLV reverse transcriptase (RNase H–) were employed to generate first-strand cDNA, and DNA polymerase I and RNase H were used for second-strand cDNA. A single adenine was then added to the 3′ ends of the double-stranded cDNA fragments that had been end-repaired using exonuclease and polymerase activities to produce blunt ends. Adapters containing hairpin loop structures were then ligated to facilitate subsequent hybridization.

Libraries were purified using an AMPure XP system (Beckman Coulter, Brea, CA, USA), enabling enrichment of cDNA fragments with insert sizes of approximately 370–420 bp. Phusion High-Fidelity DNA polymerase, universal PCR primers, and index (X) primers were used for PCR amplification. The AMPure XP system was used to purify the amplified products again, and the Agilent 2100 Bioanalyzer was used to assess library quality. Lastly, the TruSeq PE Cluster Kit v3-cBot-HS (Illumina, San Diego, CA, USA) was used to cluster index-coded libraries on a cBot Cluster Generation System as directed.

### 2.6. Bioinformatics Analysis

Sequencing data were processed and converted to sequence reads using the CASAVA (v1.8.4) software. Raw reads were cleaned through the elimination of adapter-contaminated reads, reads containing poly-N sequences, and low-quality reads (represented by >50% of bases having Qphred scores ≤ 20. For the obtained clean data, quality metrics such as Q20, Q30, and GC contents were computed. These very clean readings were used for all ensuing analyses.

DEGs between the diapause (D) and non-diapause (ND) *A. japonicus* samples were identified using DESeq in R (version 1.10.1) based on negative binomial distributions and control of the FDR by adjusting the P-values using the Benjamini–Hochberg approach and applying the criterion of adj *p* ≤ 0.05 for differential expression.

### 2.7. RT-qPCR

Primer sequences are given in Table 2. *β-Actin* represented the reference gene, as previously reported [23]. RT-qPCR was undertaken on a CFX-96 Real-Time PCR System (Bio-Rad, Hercules, CA, USA) while employing TB Green Premix Ex Taq (TaKaRa, Tokyo, Japan). Three separate biological replicates were utilized, and expression was determined with the 2^−ΔΔCT^ technique [24].

## 3. Results

### 3.1. Transcriptomics

**Figure 1:**
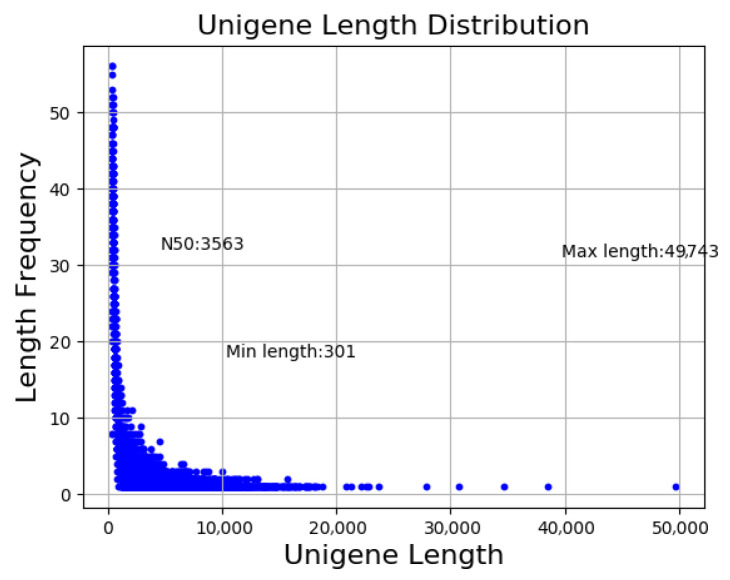
The distribution map of transcript length.

Illumina sequencing of diapause and non-diapause *A. japonicus* yielded an average of 82,748,981.33 and 94,858,824 clean reads, respectively (Figure 1). A total of 52,698 transcripts were assembled from high-quality, clean reads using Trinity. A high degree of transcriptome completeness and assembly integrity was demonstrated by the assembled transcripts’ average length of 2427 bp, N50 of 4610 bp, and 30,286 transcripts longer than 1000 bp (Table 3 and Table 4).

**Figure 2:**
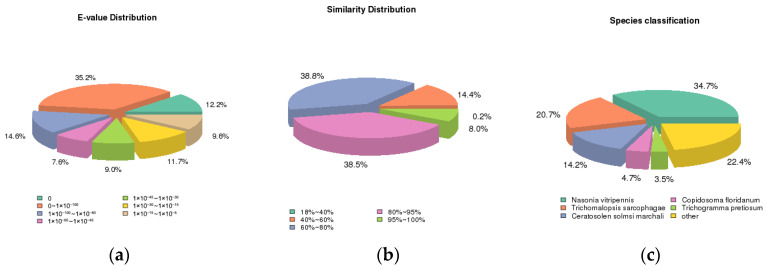
Transcript alignment results in the NR database. (**a**) Distribution of E-values after alignment in the NR database; (**b**) distribution of sequence similarities after alignment in the NR database. (**c**) Species classification plot.

Functional annotation of the assembled transcripts revealed that 15,105 transcripts were successfully annotated across various databases: NR (13,205), NT (7847), KO (5149), Swiss-Prot (9980), PFAM (11,144), GO (11,144), and KOG (5734) (Table 5). Notably, 34.7% of the annotated genes showed similarity to *Nasonia vitripennis* based on NR database results (Figure 2), a species recognized as a critical parasitoid wasp [25,26].

**Figure 3:**
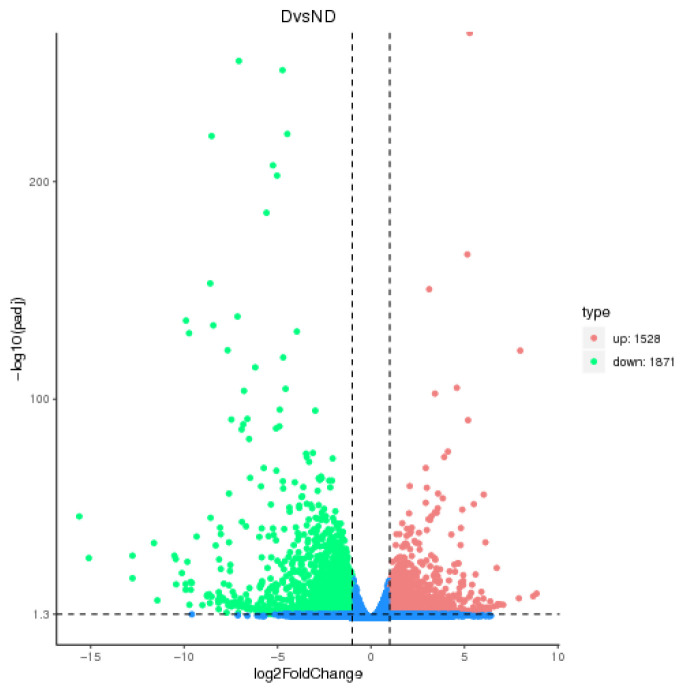
The volcano map for DEGs.

**Figure 4:**
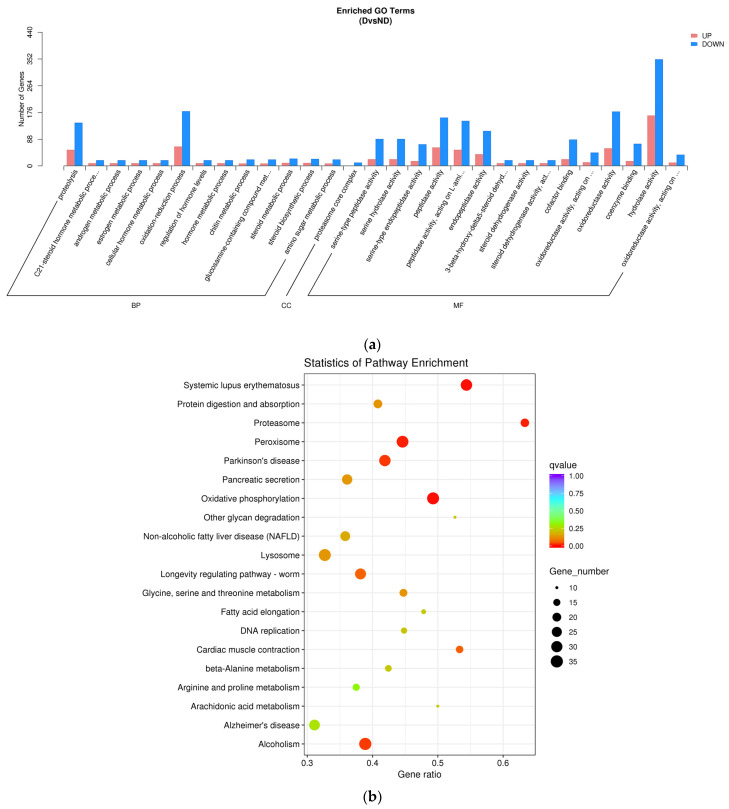
GO and KEGG analyses of DEGs between the diapause (D) and non-diapause N(D) groups. (**a**) GO enrichment bar chart; (**b**) pathway enrichment analysis bubble plot.

Overall, 3399 annotated DEGs were found, comprising 1528 upregulated and 1871 downregulated genes (Figure 3). The diapause regulator JH was linked to 10 DEGs in this dataset, including 2 upregulated and 8 downregulated genes associated with JH metabolic pathways [21,27,28]. GO and KEGG analyses were conducted to assess the DEGs’ functions. GO analysis identified five upregulated and four downregulated genes involved in hormone metabolism. KEGG pathway analysis showed that four upregulated and four downregulated DEGs were significantly enriched in the longevity-regulating pathway (Table 6; Figure 4)

### 3.2. DEG Verification

**Figure 5:**
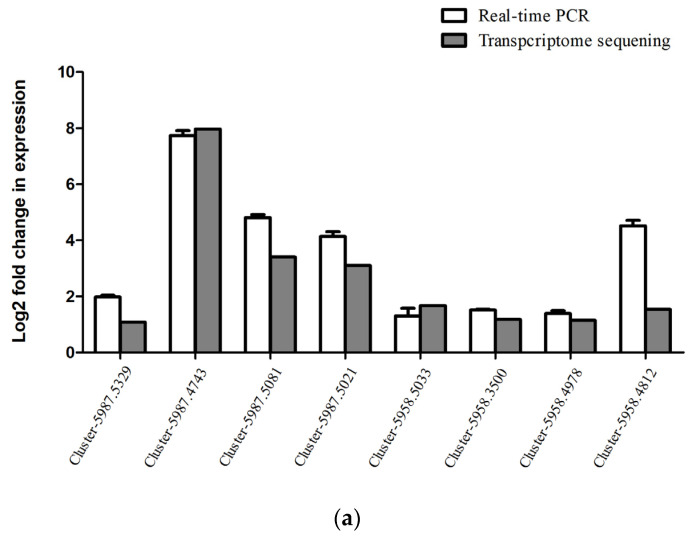

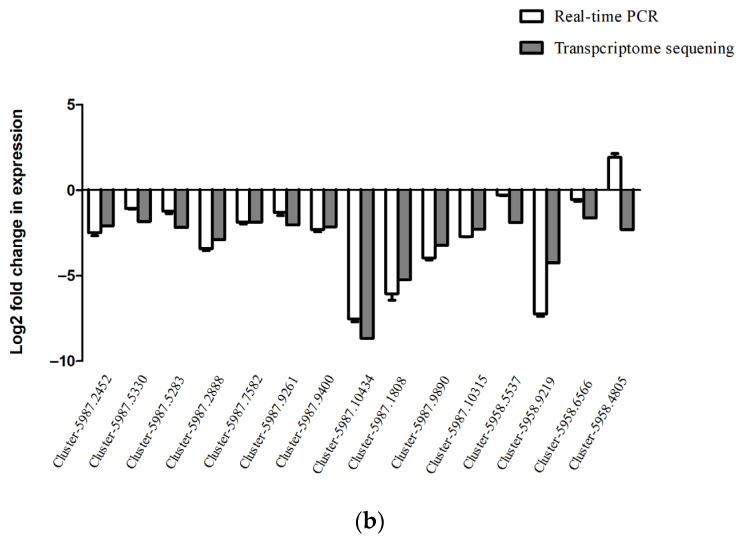
Verification of DEG expression by means of RT-qPCR. (**a**) Upregulated gene; (**b**) downregulated gene.

The levels of 23 DEGs (eight upregulated and fifteen down-regulated) with notable expression changes were verified using RT-qPCR. Except for cluster-5958.4805 (Figure 5), the levels of 22 of the 23 DEGs aligned with the RNA-seq results.

### 3.3. Global Changes in Protein Levels

After TMT labeling and HPLC separation of *A. japonicus* proteins, high-resolution LC-MS/MS analysis produced a total of 364,335 spectra. Overall, 19,099 peptides and 3112 proteins were found from 31,433 spectra that matched known entries (Table 7).

**Figure 6:**
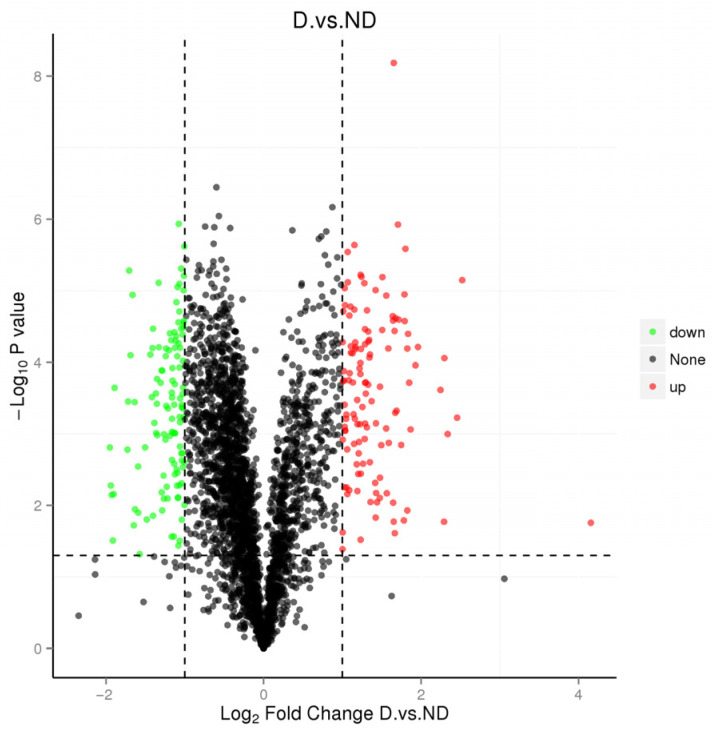
The volcano map of differential proteins.

**Figure 7:**
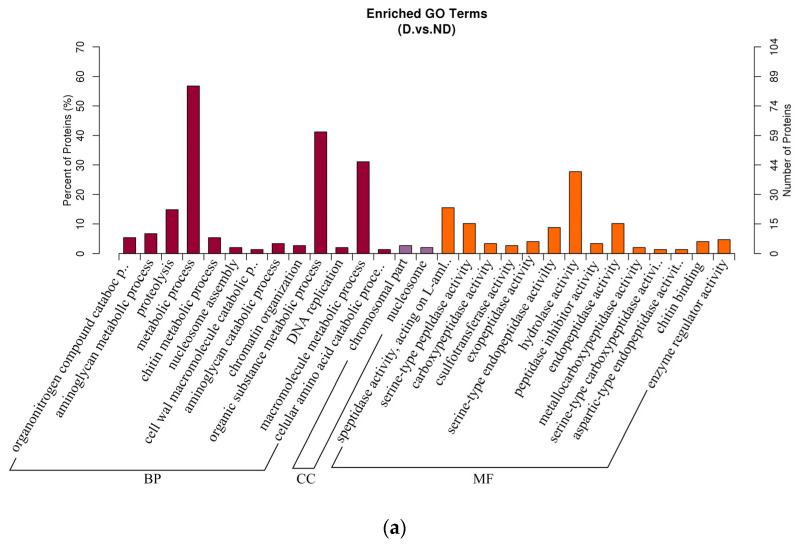

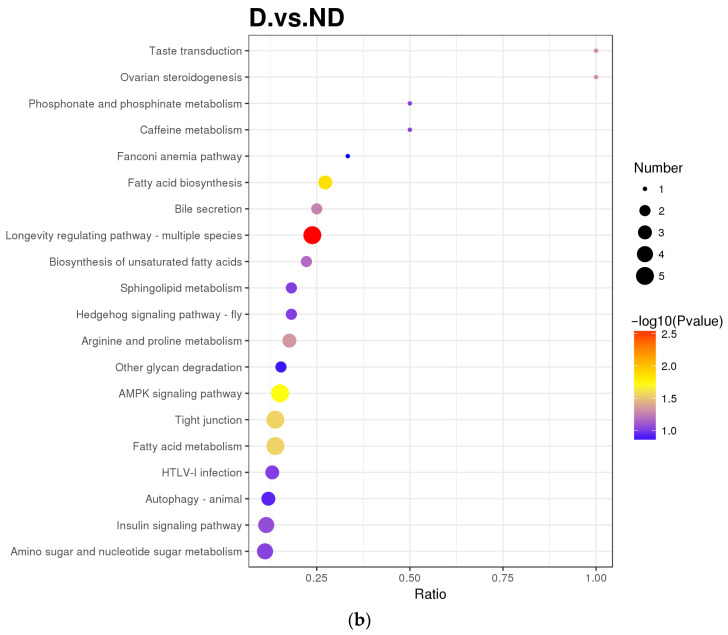
GO and KEGG analyses of DEPs between the diapause (D) and non-diapause (ND) groups. (**a**) GO enrichment bar chart; (**b**) pathway enrichment analysis bubble plot.

A volcano plot was utilized for statistical screening to find proteins with significant differential expression during diapause, with a fold-change threshold of FC > 1.9 and *p* < 0.05 (Figure 6, Table 8). A total of 12 diapause-related proteins were identified as key regulatory genes using GO and KEGG analyses (Figure 7). Among these, eight proteins were upregulated, including hemocyanin, peptidoglycan recognition protein, serine proteases, cytochrome P450 family 4, and a chitin-binding domain protein. Four proteins were down-regulated, including cell division cycle protein 123, a sulfotransferase domain-containing protein, a tudor domain protein, and major royal jelly protein (Table 8).

### 3.4. Integrative Analysis of the Proteome and Transcriptome

**Figure 8:**
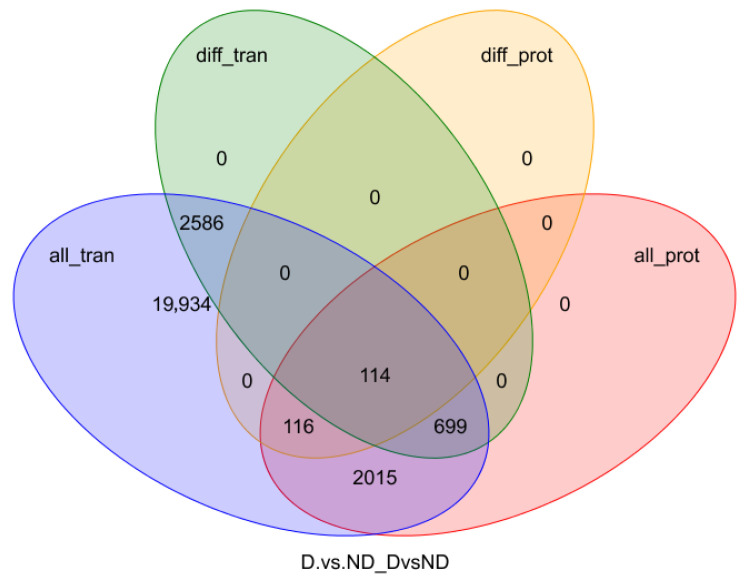
Venn diagram of the DEGs and DEPs derived from transcriptome and proteome analysis. In this context, *all_tran* denotes all genes identified by means of transcriptomic analysis, while *diff_tran* denotes the differentially expressed genes detected by means of RNA-seq. Similarly, *all_prot* represents the complete set of proteins identified in the proteomic analysis, and *diff_prot* indicates the differentially expressed proteins identified from the proteome.

**Figure 9:**
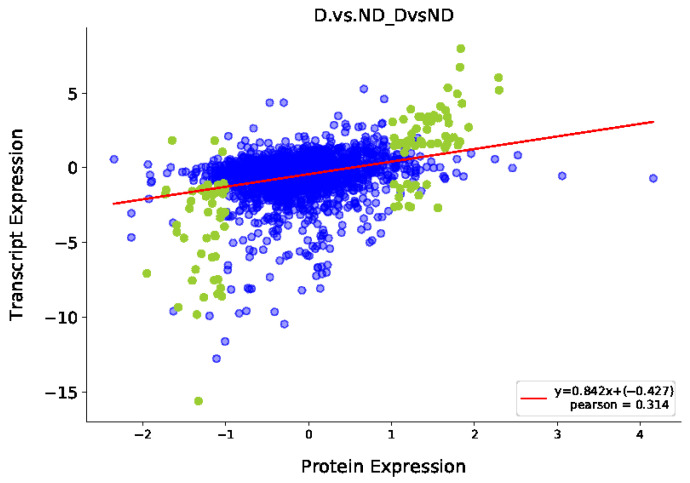
Correlation plots obtained from the transcriptome and proteome expression analyses.

**Figure 10:**
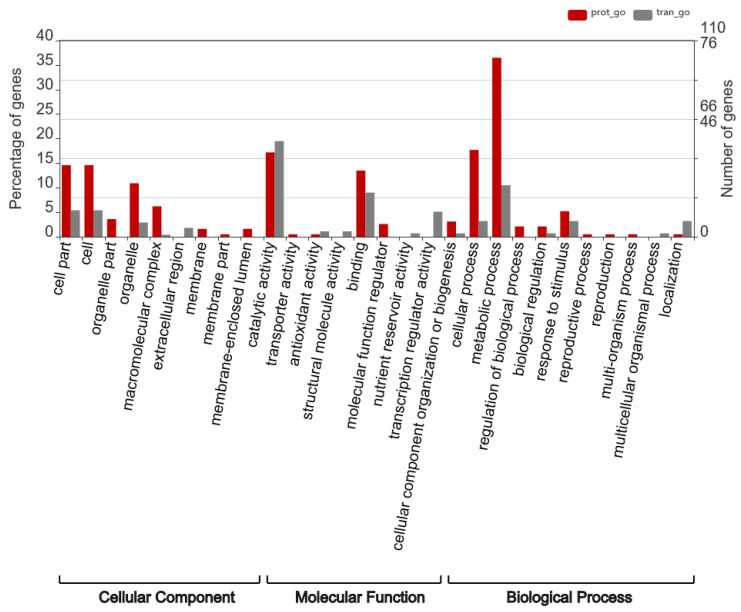
GO pathway enrichment analysis of transcriptomic and proteomic data.

Overall, 114 DEGs and DEPs were identified between the diapause and non-diapause stages of *A. japonicus* (Figure 8). To assess the concordance between transcriptional and translational changes, correlations between these DEGs and DEPs were determined, demonstrating an overall strong positive association (r = 0.842, *p* ≤ 0.05) (Figure 9). To further elucidate functional distinctions between the transcriptomic and proteomic profiles, GO enrichment analysis was conducted. The terms “cell,” “cell part,” “organelle,” and “macromolecular complex” were highly enriched cellular component terms. Enriched molecular function terms comprised “catalytic activity,” “antioxidant activity,” “binding,” and “cellular component organization or biogenesis.” Prominent biological processes were “cellular process,” “metabolic process,” “biological regulation,” “response to stimulus,” and “localization” (Figure 10).

**Figure 11:**
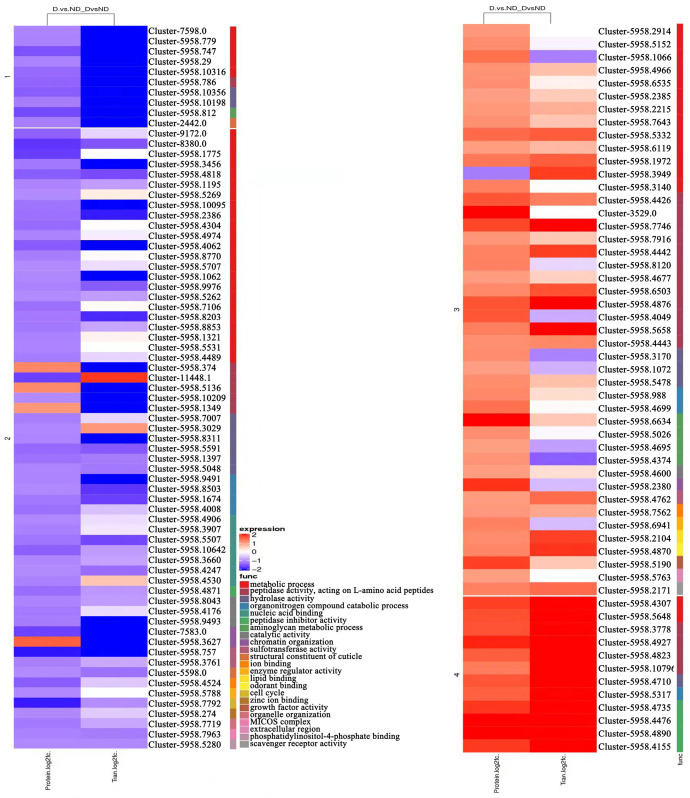
Cluster heat map of GO function enrichment. Red indicates upregulated expression, whereas blue denotes downregulated expression. Horizontal clustering reflects similarities in expression patterns at both the proteomic and transcriptomic levels, with proteins or genes within the same cluster demonstrating comparable expression trends. The individual entries shown in the figure are protein-based.

Furthermore, GO-based pathway clustering of combined transcriptomic and proteomic data indicated that upregulated DEGs and DEPs were predominantly associated with “peptidase activity,” “peptidase inhibitor activity,” “hydrolase activity,” and “lipid binding.” In comparison, downregulated genes and proteins were mainly enriched in pathways related to “aminoglycan metabolic process,” “nucleic acid binding,” “structural constituent of cuticle,” and “scavenger receptor activity” (Figure 11).

**Figure 12:**
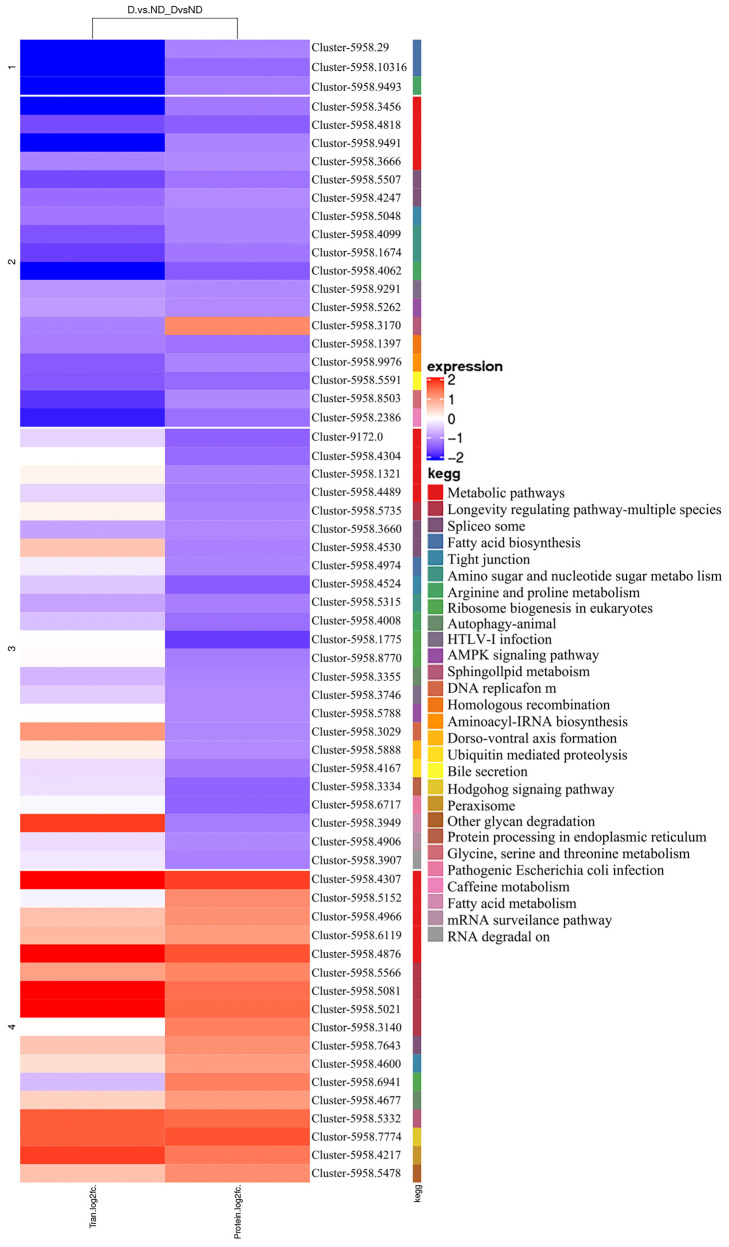
Cluster heat map of KEGG function enrichment.

KEGG analysis showed that upregulated pathways involved “sphingolipid metabolism,” “fatty acid metabolism,” “ubiquitin-mediated proteolysis,” and the “hedgehog signaling pathway.” In comparison, downregulated pathways involved “fatty acid biosynthesis,” “arginine and proline metabolism,” “spliceosome,” “fatty acid metabolism,” and “nucleotide sugar and amino sugar metabolism” (Figure 12).

To further explore key pathways regulated during diapause, we identified several candidate genes based on GO and KEGG enrichment and transcriptomic and proteomic expression levels. They are farnesol dehydrogenase, crystallin (in the longevity regulating pathway), cytochrome P450, and genes containing a forkhead-associated domain (Table 9).

## 4. Discussion

The biochemical mechanism of diapause is intricate. Diapause-related genes are expressed in response to environmental conditions. For instance, the clock gene period contributes to the regulation of final-instar larval diapause, which is regulated by short photoperiod and low temperature in female *Nasonia vitripennis* [29,30,31]. According to earlier research, *A. japonicus* displays facultative diapause of mature larvae induced by brief photoperiods (10L:14D) and a temperature of 17 °C [10]. However, the molecular pathways responsible for diapause in *A. japonicus* larvae are not known. Thus, we conducted integrated transcriptomic and proteomic analyses on *A. japonicus* samples at diapause and non-diapause stages. The four gene/protein pairs were found to be highly elevated by KEGG pathway categorization based on combined TMT proteomic and RNA-Seq transcriptomic data: Farnesol dehydrogenase (ID: 5958.4743), Crystallin alpha B (IDs: 5958.5021 and 5958.5081), and Cytochrome P450 (ID: 5958.3611). One gene/protein pair was significantly downregulated: a forkhead-associated domain-containing protein (ID: 5958.10434). These molecules are involved in juvenile hormone (JH) biosynthesis, neuropeptide hormone activation, oxidative phosphorylation, and longevity regulation pathways.

Only found in insects, JH is produced by the corpus allatum (CA) [32]. By opposing the molting hormone 20-hydroxyecdysone (20E) in larvae, JH is essential for inhibiting metamorphosis. Furthermore, it regulates key physiological processes, including oogenesis, vitellogenesis, ovulation, pheromone biosynthesis, and organ remodeling, ultimately enhancing female reproductive fitness. Consequently, JH controls developmental changes, such as diapause and reproduction [33,34,35]. For instance, low ecdysteroid titers and high JH levels induced diapause in the larvae of *Psacothea hilaris* [36]. RNA interference-mediated knockdown of *LsFoxO* in diapausing nymphs of *Laodelphax striatellus* markedly reduced JH III levels and shortened the nymphal period [21]. The CA levels of JH-synthesizing enzymes primarily determine biosynthetic activity, which in turn controls the titer of JH through a balance between its biosynthesis and breakdown [37]. A crucial enzyme in the synthesis of JH, farnesol dehydrogenase (FOLD), catalyzes the conversion of farnesyl diphosphate (FPP) to farnesoic acid, a precursor of JH synthesis. It has been proposed that FOLD limits JH production rate [38]. In our investigation, FOLD expression was significantly elevated in *A. japonicus* during diapause, suggesting its possible involvement in the diapause process.

The three primary categories of detoxification enzymes found in insects are COEs, GSTs, and P450s. Among approximately 150 detoxification-related genes identified in insects, P450s account for the largest number, roughly twice as many as GSTs or COEs [39,40]. In insect growth, development, and defense, the cytochrome P450 (CYP450) enzyme system is essential. It contributes to hormone production and to the removal of foreign substances from the body [41]. In *Trichogramma dendrolimi*, 16 CYP450 genes were differentially expressed across developmental stages, highlighting their role in maintaining cellular homeostasis under stress conditions [39]. The cytochrome P450 monooxygenase (CYP314A1) and JH acid methyltransferase (JHAMT) genes in *Laodelphax striatellus* can govern the nymphal diapause state by regulating JH titers during III and 20E in the hemolymph [20]. In our study, CYP450 expression was upregulated during diapause stages in *A. japonicus*, suggesting enhanced detoxification capacity. This upregulation likely contributes to increased stress resistance during diapause, underscoring the close relationship among CYP450s in enhancing physiological toler-ance to adverse conditions.

## 5. Conclusions

The results of the present study provide a comprehensive transcriptomic and proteomic comparison between the diapause and non-diapause stages of *A. japonicus*. We identified significant differences in the levels of genes and proteins involved in JH pathways, detoxifying enzymes, crystallins, and forkhead-associated domains during diapause, using an integrated analysis. In summary, the findings of this study provide fundamental information for understanding larval diapause and insights for future gene function research, thereby facilitating the mass rearing and preservation of *A. japonicus*.

## Figures and Tables

**Table 1:** UHPLC gradient elution program.

Time (min)	Flow Rate (nL/min)	Mobile Phase A (%)	Mobile Phase B (%)
2	600	94	6
2	600	83	17
52	600	60	40
54	600	45	55
55	600	0	100
60	600	0	100

**Table 2:** RT-qCR primers.

Primer Name	Sequence (5′ to 3′)
Cluster-5958.5329-F	TTAAATTCCCGCGCTCTC
Cluster-5958.5329-R	GGTCCCATCTTGGTCTTTC
Cluster-5958.4743-F	GTGCGACTTGAGCAATGA
Cluster-5958.4743-R	TTTCGCCATTCGTCCATAC
Cluster-5958.5081-F	GCTATGCGTCATCCAGAATC
Cluster-5958.5081-R	TGCTCGTCTTCCCTCATAA
Cluster-5958.5021-F	AAATCGCTCCATGGTCTATAAT
Cluster-5958.5021-R	GGACTTCGGTGTTGGTTT
Cluster-5958.5033-F	AGCCACTGTCTCTTCTTCT
Cluster-5958.5033-R	GGTCCAGTGAAACCCTTAAC
Cluster-5958.3500-F	CTTCACGTAGCAGTGGAATC
Cluster-5958.3500-R	CGCATCTGACTTCACGTATC
Cluster-5958.4978-F	CAAGCCCAACAACAACAAC
Cluster-5958.4978-R	GAGTCGAGAACGACATTGAC
Cluster-5958.4812-F	AGCACCACATCCATGTTTAT
Cluster-5958.4812-R	ATCTGCACCAGCAAGAAC
Cluster-5958.2452-F	GTCCCTTTGAATGTGGATTT
Cluster-5958.2452-R	GGGAGAATAATAGCAATCTCAA
Cluster-5958.5330-F	AGCCACTGTCTCTTCTTCT
Cluster-5958.5330-R	GGTCCAGTGAAACCCTTAAC
Cluster-5958.5283-F	ATTTCAGCAGCAGTCCTATG
Cluster-5958.5283-R	CTGCTGATGTACTGGCTATG
Cluster-5958.2888-F	TCAGTCCAGGTGCTGTAA
Cluster-5958.2888-R	GTGGTGTACCGAGAACATATAC
Cluster-5958.7582-F	CTACACCGGCGCAATTTA
Cluster-5958.7582-R	TTGTCAGAGCAAGATCCAAG
Cluster-5958.9261-F	ACCTGCTCCCACTATCAA
Cluster-5958.9261-R	GTCATTGCACGACCATCA
Cluster-5958.9400-F	TTCTGCTACTTGGGCTTATG
Cluster-5958.9400-R	TTGACGACCACCATGATTAG
Cluster-5958.10434-F	TTCGTCCCAGGTCTTCTT
Cluster-5958.10434-R	ATGCTGTCGAAATCGGTAAA
Cluster-5958.1808-F	CCTCGTGTTCGTGCTTAC
Cluster-5958.1808-R	GAATTGGCATGGTGAGTTTG
Cluster-5958.9890-F	CGAATTGAGCGTCCAAAGA
Cluster-5958.9890-R	CCAGCGTAAACGTCTTCAA
Cluster-5958.10315-F	CCCAAGAACAAGACGAAGA
Cluster-5958.10315-R	GGTTTCTGCTGTTTGACTTC

**Table 3:** Statistics of clean reads.

Sample	Clean Reads	Data Size (Gb)	Q30 Content (%)	GC Content (%)
D	82,748,981.33	6.21	93.79	37.08
ND	94,858,824	7.11	92.98	38.18

**Table 4:** Statistics tables of sequential assembly.

Length Range (nt)	Transcripts	
	Number	Percentage (%)
300–500 bp	7503	29.47
500–1 kbp	6735	26.45
1 k–2 kbp	4372	17.17
>2 kbp	6854	26.92
Total transcript	25,464	
N50 length of transcripts (nt)	3563	
Max length (nt)	49,743	
Min length (nt)	301	
Average length (nt)	1779	

**Table 5:** Statistics tables of gene annotations.

Database	Number of Transcripts	Percentage (%)
NR	13,205	51.85
NT	7847	30.81
KO	5149	20.22
Swiss-Prot	9980	39.19
PFAM	11,144	43.76
GO	11,144	43.76
KOG	5734	22.51
Annotated in all databases	2770	10.87
Annotated in a minimum of one database	15,105	59.31
Total Transcripts	25,464	

**Table 6:** Statistics tables of differential genes.

Gene ID	Gene Name	log2FC (D. vs. ND)	up, down (D. vs. ND)
entry 1	Data	data	
Cluster-5958.4743	Farnesol dehydrogenase	7.9696	up
Cluster-5958.5987	Fatty acyl-CoA reductase	2.4011	up
Cluster-5958.3500	steroid hormone receptor	1.1793	up
Cluster-5958.4978	steroid hormone receptor	1.1402	up
Cluster-5958.4812	neuropeptide hormone	1.5377	up
Cluster-5958.4932	essential for life-like	1.2535	up
Cluster-5958.5021	essential for life-like	3.1042	up
Cluster-5958.5081	essential for life-like	3.4098	up
Cluster-5958.4626	essential for life-like	1.5268	up
Cluster-5958.5329	heat shock 70 kDa protein	1.0837	up
Cluster-5958.5670	essential for life-like	1.1048	up
Cluster-5958.5494	transcription elongation	1.3494	up
Cluster-5958.5033	acetyl-CoA	1.6706	up
Cluster-5958.5446	Farnesol dehydrogenase	2.2548	up
Cluster-5314.0	Allatostatin	1.6238	up
Cluster-5958.3376	Lipophorin	1.0842	up
Cluster-5958.5047	Lipophorin	1.4912	up
Cluster-5958.6889	Cytochrome P450 314A1	1.0569	up
Cluster-5958.2888	Farnesol dehydrogenase	−2.8979	down
Cluster-5958.7582	L-xylulose reductase	−1.8766	down
Cluster-5958.5330	Malate dehydrogenase	−1.8331	down
Cluster-5958.5283	ubiquinol-cytochrome c reductase	−2.1812	down
Cluster-5958.10315	ubiquinol-cytochrome c reductase	−2.2849	down
Cluster-5958.2452	NADH dehydrogenase	−2.0877	down
Cluster-5958.9400	hydroxyacid oxidase 1-like	−2.1483	down
Cluster-5958.10434	Lipophorin	−8.6652	down
Cluster-5958.5537	Lipophorin	−1.8916	down
Cluster-5958.9219	Lipophorin	−4.2541	down
Cluster-5958.9890	Lipophorin	−3.2205	down
Cluster-5958.1808	NPC	−5.2372	down
Cluster-5958.6566	JH epoxide hydrolase	−1.6307	down
Cluster-5958.4805	Basic JH-suppressible protein	−2.2999	down

**Table 7:** Statistics tables of clean reads.

Total Spectra	Matched Spectrum	Peptide	Protein	All
364,335	31,433	19,099	3112	3112

**Table 8:** Statistics tables of differential protein.

Protein Number	Protein Identification	D. vs. ND *p*-Value	D. vs. ND log2FC *	D. vs. ND up, down
orf-5958.4651	Hemocyanin	0.017531	4.155729	up
orf-5958.5231	Peptidoglycan recognition protein	0.000021	2.521953	up
orf-3529.0	Serine proteases	0.000594	2.457674	up
orf-5958.3611	Cytochrome P450 family 4	0.001005	2.337397	up
orf-5958.4476	Pacifastin	0.000087	2.296085	up
orf-5958.4890	Pacifastin	0.016952	2.291289	up
orf-5958.6634	Chitin binding	0.000243	2.246311	up
orf-5958.4927	Serine proteases	0.00011	1.929038	up
orf-5958.7792	Cell division cycle protein 123	0.006963	−1.90315	down
orf-5958.757	Sulfotransferase	0.007156	−1.92721	down
orf-5958.1333	Tudor domain	0.005277	−1.93852	down
orf-5958.7647	Major royal jelly protein	0.001551	−1.95145	down

* log2FC: Convert the fold change by taking its log2.

**Table 9:** Information on diapause-related differentially expressed genes.

Gene/Protein Number (Cluster/orf)	Identification	Gene *p*-Value	Gene log2FC	Protein *p*-Value	Protein log2FC	D. vs. ND up, down
5958.4743	Farnesol dehydrogenase	2.9507 × 10^−126^	7.9696	0.0000401	1.834548	up
5958.5021	Crystallin, alpha B	1.3421 × 10^−154^	3.1042	0.000219	1.478312	up
5958.5081	Crystallin, alpha B	2.1821 × 10^−106^	3.4098	0.009065	1.440745	up
5958.3611	Cytochrome P450	4.7981 × 10^−77^	3.9116	0.00000599	1.232454885	up
5958.10434	Forkhead-associated domain	1.34 × 10^−12^	−8.6652	0.00807	−1.26483	down

|log2(FoldChange)| > 1 and padj < 0.05.

## Data Availability

The data that support the findings of this study are available from the corresponding author upon reasonable request.
